# Notes

**Published:** 1895-11

**Authors:** 


### Notes.

---

Pædiatrics is the name of a new journal devoted to diseases of children. It is published semimonthly in New York. Dr. Dillon Brown, owner; Dr. George Carpenter, of London, editor.

We take pleasure in announcing that Dr. Frederick Holme Wiggin has been reappointed by the Commissioners of Charities and Corrections as visiting Gynæcologist to the New York City Hospital (Blackwell's Island).

At the last meeting of the Tri-State Medical Society of Iowa, Illinois and Missouri, the following officers were elected: President, Dr. Robert H. Babcock, Chicago; First Vice President, Dr. A. H. Cordier, Kansas City; Second Vice President, Dr. W. A. Todd, Chariton, Iowa; Treasurer, Dr. C. S. Chase, Waterloo, Iowa; Secretary, Dr. G. W. Cale, St. Louis. The next meeting will be held in Chicago the first Tuesday, Wednesday and Thursday in April, 1896.

W. B. Saunders, 925 Walnut Street, Philadelphia, the well-known publisher of medical text-books, advises us that he will have ready by January 1st, '96, "The American Yearbook of Medicine and Surgery," edited by George W. Gould, M. D. It is the aim of the publisher to place before the physician, in convenient form, *an epitomization of current journalistic literature by persons competent to pronounce upon the value of a method of treatment.* It is the special purpose of the editor, whose experience peculiarly qualifies him for the preparation of this work, not only to review the contributions to American journals, but also the methods and discoveries reported in the leading medical journals of Europe, thus enlarging the survey and making the work characteristically international. "The Yearbook of Medicine and Surgery" will be issued annually hereafter.

